# Prevalence and factors associated with cancer-related fatigue in Swiss adult survivors of childhood cancer

**DOI:** 10.1007/s11764-023-01413-1

**Published:** 2023-06-13

**Authors:** Tomáš Sláma, Fabiën N. Belle, Sven Strebel, Salome Christen, Eva Hägler-Laube, Jochen Rössler, Claudia E. Kuehni, Nicolas X. von der Weid, Christina Schindera

**Affiliations:** 1 Childhood Cancer Research Group, Institute of Social and Preventive Medicine, University of Bern, Bern, Switzerland; 2 Graduate School for Cellular and Biomedical Sciences, University of Bern, Bern, Switzerland; 3 Center for Primary Care and Public Health (Unisanté), University of Lausanne, Lausanne, Switzerland; 4 Graduate School for Health Sciences, University of Bern, Bern, Switzerland; 5 CANSEARCH Research Platform in Pediatric Oncology and Hematology, Department of Pediatrics, Gynecology and Obstetrics, University of Geneva, Geneva, Switzerland; 6 Department of Health Sciences and Medicine, University of Lucerne, Lucerne, Switzerland; 7 Department of Internal Medicine, Cantonal Hospital Baden, Baden, Switzerland; 8 Pediatric Oncology, Inselspital, Bern University Hospital, University of Bern, Bern, Switzerland; 9 Division of Pediatric Oncology/Hematology, University Children’s Hospital Basel, University of Basel, Basel, Switzerland

**Keywords:** Fatigue, Childhood cancer, Survivors, Questionnaires, Late effects

## Abstract

**Purpose:**

Reported prevalence of cancer-related fatigue (CRF) among childhood cancer survivors (CCS) varies widely, and evidence on factors associated with CRF among CCS is limited. We aimed to investigate the prevalence of CRF and its associated factors among adult CCS in Switzerland.

**Methods:**

In a prospective cohort study, we invited adult CCS who survived at least 5 years since last cancer diagnosis, and were diagnosed when age 0–20 years and treated at Inselspital Bern between 1976 and 2015 to complete two fatigue-measuring instruments: the Checklist Individual Strength subjective fatigue subscale (CIS8R; increased fatigue 27–34, severe fatigue ≥ 35) and the numerical rating scale (NRS; moderate fatigue 4–6, severe fatigue 7–10). We collected information about previous cancer treatment and medical history, and calculated *β* coefficients for the association between CIS8R/NRS fatigue scores and potential determinants using multivariable linear regression.

**Results:**

We included 158 CCS (participation rate: 30%) with a median age at study of 33 years (interquartile range 26–38). Based on CIS8R, 19% (*N* = 30) of CCS reported increased fatigue, yet none reported severe fatigue. CRF was associated with female sex, central nervous system (CNS) tumors, sleep disturbance, and endocrine disorders. Lower CRF levels were observed among CCS age 30–39 years compared to those younger.

**Conclusions:**

A considerable proportion of adult CCS reported increased levels of CRF.

**Implications for Cancer Survivors:**

CCS who are female and < 30 years old, have a history of CNS tumor, report sleep disturbance, or have an endocrine disorder should be screened for CRF.

**Supplementary Information:**

The online version contains supplementary material available at 10.1007/s11764-023-01413-1.

## Background

Cancer-related fatigue (CRF) is a common and disturbing late effect in cancer patients and survivors which is often underdiagnosed and undertreated [1, 2]. CRF is defined as “a distressing, persistent, subjective sense of physical, emotional, and/or cognitive tiredness or exhaustion related to cancer or cancer treatment that is not proportional to recent activity and interferes with functioning” according to the National Comprehensive Cancer Network (NCCN) [3]. CRF usually diminishes in the first year after treatment completion, yet a previous study has shown that 24% of childhood cancer survivors continued to experience CRF up to two decades after cancer diagnosis [4]. The etiology of CRF is multi-factorial and poorly understood [1]. Biological, demographic, psychosocial, and behavioral factors influence the development of CRF among cancer patients and survivors [1]. For this reason, there is no “gold standard” of treatment; however, several approaches, such as exercise, psychosocial interventions, and mind–body interventions, showed positive effects reducing fatigue [1]. To accurately identify fatigued survivors, implementing regular screening for CRF in long-term follow-up care of childhood and adolescent cancer survivors (CCS) is recommended [5].

Reported prevalence of CRF in CCS varies widely in the literature—from 0 to 62% [6]. Variability in prevalence is due to differences in study designs, methodology, and fatigue-measuring instruments. Until 2020, there was no unified recommendation regarding which fatigue-measuring instrument to use in CCS [5]. Therefore, a large number of instruments including the Checklist Individual Strength (CIS), or the numerical rating scale (NRS), were in use. Recent guidelines for surveillance of CRF among childhood, adolescent, and young adult cancer survivors by the International Guideline Harmonization Group (IGHG) show knowledge gaps about factors associated with CRF for this population [5]. Many treatment-related, clinical, and sociodemographic factors have been studied as contributors of cancer-related fatigue in CCS, such as anxiety, pain, and educational level [5, 7]. However, psychological distress is the only factor with high quality of evidence available [5]. Other associated factors, such as late effects, pain, older age, radiotherapy, and sleep problems, have moderate or low levels of evidence [5]. It is likely that the etiology of cancer-related fatigue is multifactorial [1, 8, 9], and sufficient evidence on CRF prevalence and factors associated with it is crucial for establishing and updating clinical guidelines on CRF in CCS, such as those from the IGHG. Therefore, in this study, we aimed to evaluate the prevalence of CRF and factors associated with CRF among CCS.

## Methods

### Study design and population

Our study is part of the CardioOnco study investigating cardiovascular health among adult CCS set up within routine care in a cardio-oncology clinic. Detailed information about the CardioOnco study design is available [10]. It was initiated in 2016 as a single-center study involving Pediatric Hematology and Oncology and Pediatric and Adult Cardiology at the University Hospital Bern, Inselspital, in Switzerland. The study invited all CCS diagnosed with childhood cancer since 1976, who survived at least 5 years since diagnosis, were treated at the University Children’s Hospital Bern with any chemotherapy and/or heart-relevant radiotherapy, were older than age 18 years at the time of study, and who were registered in the Swiss Childhood Cancer Registry (ChCR). The ChCR includes all patients in Switzerland diagnosed before the age of 20 years with any childhood cancer coded according to the International Classification of Childhood Cancer Third Edition (ICCC-3) [11, 12]. We excluded survivors who were treated with surgery only and/or radiotherapy other than heart-relevant radiotherapy. We invited eligible survivors identified by the ChCR by post to visit the cardio-oncology clinic. During clinic visits, we took medical history, performed physical examinations and echocardiograms, and counselled survivors about their cardiovascular health. A few hours after the visit, survivors received an online survey link via email. This online survey includes questionnaires on fatigue, physical activity, nutrition, and quality of life.

### Population characteristics

#### Sociodemographic variables

When taking medical history during the visit, we collected data on age at study, marital and employment status, and parenting children.

#### Lifestyle variables

During the visit, we asked survivors about their smoking status. We also performed anthropometry to obtain survivors’ body mass index (BMI) and waist–hip ratio. We defined and classified both variables according to World Health Organization cutoff points [13, 14].

#### Clinical variables

In March 2016, ChCR provided data about eligible survivors, such as age at cancer diagnosis, time since diagnosis, cancer diagnosis, and history of relapse. We asked survivors during clinic visits or later extracted from medical records whether he/she has had second primary malignancy, sleep disturbance, endocrine disorders, and antidepressant use as a proxy for depression. We also asked survivors about possible sleep disturbance using three yes–no questions: “Do you generally have problems falling asleep?”; “Do you generally wake up several times during the night?”; and “Do you generally have problems sleeping through the night?”. We defined sleep disturbance as answering “yes” to one or more questions similar to previous studies [15].

#### Treatment-related variables

From medical records, we collected information on anthracyclines (including cumulative doses), alkylating agents, heart-relevant and/or cranial radiotherapy (CRT; including cumulative doses), and hematopoietic stem cell transplantation (HSCT). Heart-relevant radiotherapy was defined as any therapeutic exposure of the chest, abdomen, spine (thoracic or whole), and total body irradiation (TBI) [16]. If a survivor received TBI, we added the dose to heart-relevant radiotherapy and CRT. We also collected information about intrathoracic surgery and cancer treatment duration.

### Fatigue measuring instruments

We used two different measuring instruments to assess CRF (see Supplementary Materials 1 and 2). On the day of their clinic visit, CCS participants were invited to take the online questionnaire, which included both instruments. The Checklist Individual Strength subjective fatigue subscale (CIS8R) is an 8-item self-reporting instrument. It is one of four subscales of the Checklist Individual Strength instrument introduced by Vercoulen et al. in 1994 which is a multidimensional measure of severity and behavioral consequences of fatigue [17–19]. Each item is scored on a 7-point Likert scale (1 = “yes, that is true”; 7 = “no, that is not true”) [20]. Reversed scoring is applied to some items [21]. Statements of the questionnaire refer to aspects of fatigue experienced during the previous 2 weeks; higher scores indicate higher degree of fatigue [20]. We chose to analyze the results of CIS8R since it is a reliable and validated instrument for assessing CRF [21]. The psychometric properties of the CIS are good among adult CCS population—it correlates highly with other fatigue measures—and the CIS8R especially showed excellent internal consistency [22]. The range for CIS8R is 8–56 [23]. Scores between 27 and 34 were defined as increased fatigue and a score of 35 or higher as severe fatigue [21, 24]. As a second instrument, we used the NRS. We asked survivors “How intense/strong is your fatigue at the moment?” We asked participants to mark the point best representing perception of their current fatigue state on a scale from 0 to 10. We graded fatigue as moderate with scores 4 to 6 and as severe with scores 7 to 10 [25].

### Statistical analysis

We calculated the prevalence of increased and severe CRF based on CIS8R and moderate and severe CRF based on NRS, overall and stratified by sex. We then fitted univariable linear regression models to identify associations between higher fatigue scores and sociodemographic, lifestyle, clinical, and treatment-related characteristics of the study population. In this model, we selected a priori all possible factors associated with CRF known from the literature [5] which were available in our dataset. We then included variables associated with increased CRF scores in at least one of the two fatigue instruments at *p* < 0.1 in the multivariable analysis. To avoid overfitting, we performed a backward selection of variables for the multivariable analysis using corrected Akaike’s information criterion (AICc) [26]. All *p* values are two-sided; we considered *p* < 0.05 statistically significant. We calculated *p* values using likelihood-ratio tests. All analyses were performed using Stata software, version 16.1 (StataCorp. 2019, Stata Statistical Software: Release 16, College Station, TX: StataCorp LLC). We used the coefplot command for graphically presenting our multivariable linear regression analysis [27].

## Results

### Characteristics of study population

**Fig. 1:**
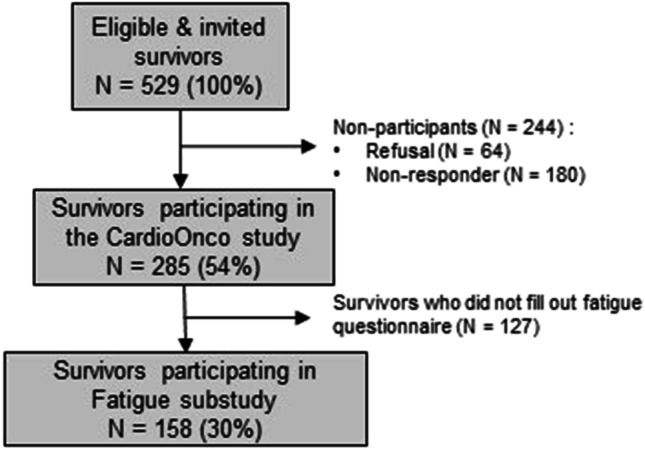
Flowchart of the study population. *N*, number

Between March 2016 and September 2021, we invited 529 eligible CCS to the CardioOnco study. Of those, 285 (54%) participated; 64 (12%) refused to participate; and 180 (34%) did not respond. Of all invited survivors, 158 (30%) filled out questionnaires that included both fatigue-measuring instruments (Fig. 1). We included slightly more females (51%; Table 1). The median age at time of study was 33 years (interquartile range [IQR] 26–38), and the median age at diagnosis was 7 years (IQR 2–13). The most frequent cancer diagnoses were leukemia (37%), lymphoma (22%), and malignant bone tumors (11%). Thirteen percent had experienced a relapse. Criteria for sleep disturbance were fulfilled among 28% of CCS, while endocrine disorders were observed in 22%. Radiotherapy had been administered to 35% of CCS. Twenty-nine percent of CCS received heart-relevant radiotherapy with a median cumulative dose of 25.5 Gray (Gy; IQR 18–38.5); 15% received CRT with a median cumulative dose of 33 Gy (IQR 18–51.6); and 9% received both. In Supplementary Table 1, we show basic characteristics of non-participating and participating CCS who did not complete fatigue-measuring instruments. The comparison of participants and non-participants of our study shows that age at study, chemotherapeutic treatment, and HSCT differed. Younger CCS were more reluctant to participate.

**Table 1:** Sociodemographic, lifestyle, clinical, and treatment-related characteristics of participating adult survivors of childhood cancer

	Total *N* = 158 (%)^a^	Males *N* = 78 (%)^a^	Females *N* = 80 (%)^a^
Sociodemographic characteristics			
Age at study, years, median [IQR]	33 [26-38]	33 [27-38]	33 [24.5–38]
< 30	59 (37%)	25 (32%)	34 (43%)
30–39	68 (43%)	38 (49%)	30 (38%)
≥ 40	31 (20%)	15 (19%)	16 (20%)
Married, yes	53 (34%)	30 (38%)	23 (29%)
Children, yes	42 (27%)	22 (28%)	20 (25%)
Employment, yes	126 (80%)	69 (88%)	57 (71%)
Lifestyle characteristics			
Smoking currently, yes	24 (15%)	13 (17%)	11 (14%)
Body mass index, kg/m^2^, median [IQR]^b^	23.5 [21.3–26.4]	24.1 [22.1–26.9]	23.2 [20.7–25.9]
Underweight	4 (3%)	-	4 (5%)
Normal weight	95 (60%)	47 (60%)	48 (60%)
Overweight	43 (27%)	25 (32%)	18 (23%)
Obese	16 (10%)	6 (8%)	10 (13%)
Waist-hip ratio, median [IQR]^c^	0.83 [0.78–0.91]	0.87 [0.81–0.93]	0.79 [0.76–0.86]
No abdominal obesity	97 (61%)	44 (56%)	53 (66%)
Abdominal obesity present	46 (29%)	26 (33%)	20 (25%)
* Missing measurements*	15 (9%)	8 (10%)	7 (9%)
Clinical characteristics			
Age at diagnosis, years, median [IQR]	7 [2-13]	6 [2-13]	9 [9-13]
Time since diagnosis, years, median [IQR]	25 [18-32]	26 [19-34]	25 [17.5–31]
ICCC-3 cancer diagnoses			
I Leukemias	58 (37%)	24 (31%)	34 (43%)
II Lymphomas	34 (22%)	22 (28%)	12 (15%)
III CNS tumors	8 (5%)	5 (6%)	3 (4%)
IV Neuroblastoma	6 (4%)	3 (4%)	3 (4%)
V Retinoblastoma	3 (2%)	-	3 (4%)
VI Renal tumors	11 (7%)	5 (6%)	6 (8%)
VII Hepatic tumors	2 (1%)	2 (3%)	-
VIII Malignant bone tumors	18 (11%)	8 (10%)	10 (13%)
IX Soft tissue sarcomas	10 (6%)	4 (5%)	6 (8%)
X Germ cell tumors	1 (1%)	-	1 (1%)
XI–XII Other tumors	7 (4%)	5 (6%)	2 (3%)
*Total IV*–*XII*	58 (37%)	27 (35%)	31 (39%)
Relapse, yes	21 (13%)	15 (19%)	6 (8%)
Second primary malignancy, yes	8 (5%)	3 (4%)	5 (6%)
Sleep disturbance^d^, yes	44 (28%)	15 (19%)	29 (36%)
Endocrine disorders^e^, yes	35 (22%)	17 (22%)	18 (23%)
Intake of antidepressants, yes	11 (7%)	4 (5%)	7 (9%)
Treatment-related characteristics			
Anthracyclines, yes	107 (68%)	59 (76%)	48 (60%)
No	51 (32%)	19 (24%)	32 (40%)
> 0 and < 250 mg/m^2^	67 (42%)	37 (47%)	30 (38%)
≥ 250 mg/m^2^	40 (25%)	22 (28%)	18 (23%)
Alkylating agents, yes	96 (61%)	51 (65%)	45 (56%)
Radiotherapy, yes	56 (35%)	29 (37%)	27 (34%)
Heart-relevant radiotherapy, yes^f^	46 (29%)	23 (29%)	23 (29%)
> 0 and < 15 Gy	9/46 (6%)	5/23 (6%)	4/23 (5%)
≥ 15 and < 35 Gy	20/46 (13%)	10/23 (13%)	10/23 (13%)
≥ 35 Gy	17/46 (11%)	8/23 (10%)	9/23 (11%)
Cranial radiotherapy, yes	24 (15%)	17 (22%)	7 (9%)
> 0 and < 35 Gy	12/24 (8%)	10/17 (13%)	2/7 (3%)
≥ 35 Gy	12/24 (8%)	7/17 (9%)	5/7 (6%)
Radiotherapy relevant to both brain and heart^g^	14 (9%)	11 (14%)	3 (4%)
Hematopoietic stem cell transplantation, yes	9 (6%)	8 (10%)	1 (1%)
Intrathoracic surgery, yes	19 (12%)	6 (8%)	13 (16%)
Treatment era			
1976 to 1985	35 (22%)	22 (28%)	13 (16%)
1986 to 1995	62 (39%)	27 (35%)	35 (44%)
1996 to 2005	44 (28%)	20 (26%)	24 (30%)
2006 to 2015	17 (11%)	9 (12%)	8 (10%)
Duration of treatment, months, median [IQR]^h^	12 [6-31]	10 [5-29]	15 [6-31]
≤ 1 year	79 (50%)	42 (54%)	37 (46%)
> 1 year	79 (50%)	36 (46%)	43 (54%)

*N*, number; *IQR*, interquartile range; *ICCC-3*, International Classification of Childhood Cancer 3rd edition; *CNS*, central nervous system

^a^Column percentages are given

^b^Body mass index was classified as underweight (< 18.5 kg/m^2^), normal weight (≥ 18.5 – < 25 kg/m^2^), overweight (≥ 25 – < 30 kg/m^2^), and obese (≥ 30 kg/m^2^)[13]

^c^Abdominal obesity was defined according to WHO cutoff point as waist–hip ratio ≥ 0.90 cm in men and ≥ 0.85 cm in women[14]

^d^Sleep disturbance deemed to be present if survivors answered “yes” to one or more of the following questions: “Do you have problems falling asleep?”, “Do you have problems sleeping through the night?”, or “Do you wake up multiple times during the night?”

^e^Including hyperthyroidism, hypothyroidism, diabetes mellitus, diabetes insipidus, growth hormone deficiency, and any other hormonal disorder

^f^According to the Children’s Oncology Group Guidelines Version 5.0 (i.e., chest, abdomen, whole or thoracic spine, total body irradiation) [16]

^g^Including survivors who received total body irradiation

^h^Including treatment of primary cancer and relapses

### Prevalence and CRF severity

Based on CIS8R, 30 (19%; 95% confidence interval [CI] 13–26%) CCS had increased fatigue and no survivor had severe fatigue. In the whole cohort, median CIS8R scores were 19 (IQR 14–25); 16 (IQR 13–21) for males; and 22 (IQR 16–26) for females. Based on NRS, we identified 33 survivors (21%; 95% CI 15–28%) as moderately fatigued and 37 (23%; 95% CI 17–31%) survivors as severely fatigued. NRS median scores were 3.1 (IQR 1.8–6.4) for the whole cohort, 2.2 (IQR 1.2–3.9) for males, and 5.3 (IQR 2.7–7.4) for females. Out of the 30 CCS identified by CIS8R with increased (or severe) CRF, 27 CCS were also identified by NRS as moderately or severely fatigued (Supplementary Figure 1).

### Factors associated with increased CRF

**Fig. 2:**
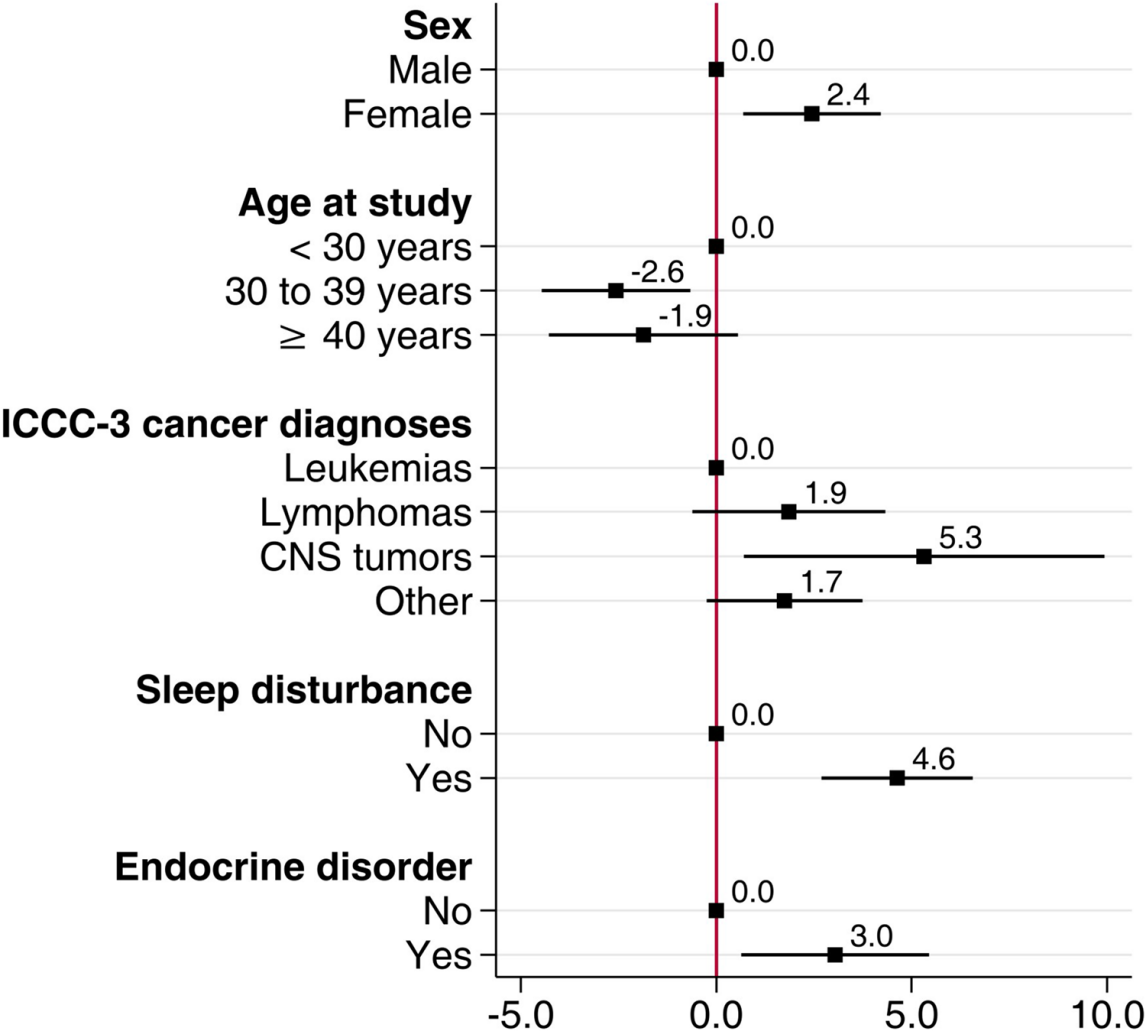
Forest plot of *β* coefficients with 95% confidence intervals retrieved from multivariable linear regression showing the association between CRF levels measured by CIS8R and sex, age at study, ICCC-3 cancer diagnosis, sleep disturbance, and endocrine disorders. Higher coefficients represent stronger associations of variables with increased CRF levels. Except for ICCC-3 cancer diagnoses, all *p* values were < 0.05. Abbreviations: CIS8R, Checklist Individual Strength subjective fatigue subscore; CRF, cancer-related fatigue; ICCC-3, International Classification of Childhood Cancer 3rd edition

We found that female sex (*β* coefficient [*β*] 2.4; 95% CI 0.7–4.2), central nervous system (CNS) tumors (*β* 5.3; 95% CI 0.7–9.9), sleep disturbance (β 4.6; 95% CI 2.7–6.6), and endocrine disorders (*β* 3.0; 95% CI 0.6–5.4) were associated with more CRF in CIS8R in multivariable linear regression analysis (Fig. 2; Supplementary Table 2). Survivors with an age at study between 30 and 39 years (*β* − 2.6; 95% CI − 4.5 to − 0.7) experienced less CRF as measured by CIS8R than younger CCS. We observed similar associations in the model based on NRS scores (Supplementary Table 2). We present results of univariable linear regression in Supplementary Tables 3A–C.

## Discussion

We found that about one-fifth of CCS reported increased CRF many years after cancer diagnosis, while none reported severe CRF. Female survivors, survivors of CNS tumors, and those with sleep disturbance or endocrine disorders had more CRF than others. Older age at study was associated with lower levels of CRF compared with those aged < 30 years.

Since study populations differ by age, cancer treatments and diagnoses, and outcome definition, reported prevalence of CRF among adult CCS varies widely [6]. Comparing results from current studies is also difficult because up to eight different questionnaires were used in CRF prevalence studies [6]. Similar to our study, Lopez-Guerra et al. found no participant reported severe CRF [28], yet only included 17 long-term survivors of Ewing sarcoma with a median age at study of 19 years. Calaminus et al. found 4% of participants severely fatigued in a cohort of 725 Hodgkin lymphoma survivors with median age at study of 28 years [29]. However, in large studies unrestricted by including survivors of only one cancer diagnosis, the prevalence of severe CRF was higher [6]. In the North America-based Childhood Cancer Survivor Study (CCSS), 14% of 1821 adult CCS (mean age at study: 35 years) were identified as severely fatigued using the Functional Assessment of Chronic Illness Therapy-Fatigue (FACIT-F) instrument [30]. In the Dutch CCS study (DCCSS-LATER), 26% of 2516 adult CCS (median time since diagnosis: 22 years) were identified as severely fatigued using the Short Fatigue Questionnaire (SFQ) [4]. In the British CCS study (BCCSS), 33% of 9920 adult CCS (median age at study: 33 years) were identified as severely fatigued using the Short Form 36 Health-status Survey (SF-36) [31]. Since these studies used different fatigue-measuring instruments, reported prevalences fluctuated strongly. The fluctuations emphasize the need for a harmonized assessment of CRF among CCS to better understand CRF prevalence among adult CCS.

Our study showed female sex was associated with higher CRF levels. This has been described before in CCS, but the overall level of evidence is very low according to IGHG criteria [5, 32–38]. In the general population, females also more often report CRF, yet the reason is unclear [39, 40]. It does not appear solely attributable to health conditions that have a higher prevalence in women and are known to be associated with fatigue (e.g., depression) [41]. We further saw higher CRF levels among CNS tumor survivors when compared with survivors of leukemia. While the recently published article by van Deuren et al. reports statistically significant association between CRF and previous diagnosis of a CNS tumor in adult CCS, Mulrooney et al. report association which is not statistically significant, and Langeveld et al. report no association [4, 35, 36].

Sleep disturbance and endocrine disorders were associated with increased CRF in our study. Meeske et al. also reported a significant association between sleep disturbance and CRF among 161 adult survivors of acute lymphoblastic leukemia (OR = 6.15; 95% CI 2.3–16.2) [42]. Since clustering of CRF and sleep problems is well documented among adult cancer survivors, the low level of evidence for associations between sleep disorders and CRF among adult CCS that Christen et al. reported is surprising [5, 43–46]. As for endocrine disorders, the literature differs on the spectrum of endocrine disorders considered. While Mulrooney et al. and Hamre et al. showed no association of hypothyroidism with CRF, Sato et al. showed an association of endocrine abnormality with CRF among CCS [33, 35, 47]. Among endocrine disorders that we assessed in our study, diabetes mellitus and hypothyroidism had the strongest correlation with CRF in subsequent multivariable linear regression models replacing the general endocrine disorder variable with individual endocrine disorders that we performed ex post (Supplementary Tables 4A–F).

In our study, age at study was also associated with CRF severity. The literature on the topic of age at study is conflicting. When looking at the available studies, it is important to differentiate whether this variable was assessed as continuous or categorical. As for age at study as a continuous variable, Hamre et al. and Johansdottir et al. showed a weak but statistically significant positive association of older age at study with CRF (OR 1.04; 95% CI 1.0–1.1 and 1.08; 95% CI 1.01–1.16 respectively) [34, 48]. Two studies showed no significant association of older age with CRF which is in line with the finding of our univariable model [33, 36]. The weak association with age at study as a continuous variable might be caused by different levels of CRF expressed over the course of life. As we showed in our multivariable analysis, CRF follows a U-shaped pattern across the three age categories with lowest CRF among CCS aged 30–39 years. Paradoxically, the recent study by van Deuren et al. showed an upside-down U-shaped pattern across age categories in terms of CRF prevalence [4]. This paradox could be caused by different study designs since van Deuren et al. assessed prevalence of CRF in a national cohort whereas our study is a single-center study of survivors at a cardio-oncology clinic. Further studies are needed to clarify the course of CRF over lifetime.

### Strengths and limitations

Our study is the first study on prevalence and factors associated with CRF among Swiss adult CCS. Since the study setting allowed gathering high-quality and reliable data on treatment exposures, current medical histories, anthropometry, and CCS lifestyles, the study setting was valuable. It allowed analyzing details from a spectrum of possible factors associated with CRF when compared with studies with only self-reported data. However, the study setting was also a limitation since the main study interest was assessing cardiovascular health during outpatient clinic visits. For this reason, survivors had to accept the invitation and attend the outpatient clinic after which they received the fatigue questionnaire—a potentially significant obstacle for severely fatigued survivors and likely contributor to the relatively low participation rate of 30%. For this reason, severely fatigued CCS are possibly underrepresented in our study. However, when comparing participants with those who took part in the CardioOnco study but did not fill out fatigue-measuring instruments in the questionnaire, we can see that there are no significant differences between these two populations. The study design possibly introduces further selection bias, since CCS treated with surgery only were excluded, yet current research shows no effect of surgical treatment on CRF among CCS [5]. CCS with shorter time since diagnosis, e.g., of 5 to 9 years, were less represented in our cohort than those with longer time since diagnosis. This may have underrepresented the presented CRF prevalence since the risk for CRF decreases with time since diagnosis [5].

## Conclusion

We showed a substantial proportion of survivors suffer from increased levels of CRF that might interfere with their daily functioning. We identified demographic and clinical factors associated with increased CRF which could help to better identify CCS at risk for CRF. Identifying CRF-associated factors is important for the development of CRF surveillance guidelines and ensuring better tailored follow-up care of CCS. In summary, healthcare professionals need to be aware of the increased risk of CRF among adult survivors of childhood cancer and should actively screen CCS, particularly female survivors, < 30 years old, CNS tumor survivors, and survivors with sleep disturbance or endocrine disorders.

### Supplementary Information

Below is the link to the electronic supplementary material.

Supplementary file1 (DOCX 158 KB)

## Data Availability

Upon reasonable request, the corresponding author can furnish datasets generated and/or analyzed during the current study.
