# Supplementary material for: Prevalence and factors associated with cancer-related fatigue in Swiss adult survivors of childhood cancer
Source: J Cancer Surviv. 2023 Jun 13;18(1):135–43. doi: 10.1007/s11764-023-01413-1 (PMC10866786; doi:10.1007/s11764-023-01413-1)
Supplement: Supplementary file 1 — Supplementary file1 (DOCX 158 KB) [file 11764_2023_1413_MOESM1_ESM.docx]

**Prevalence and factors associated with fatigue in Swiss adult survivors of childhood cancer**

**Journal of Cancer Survivorship**

Tomáš Sláma^1,2^, Fabiën N Belle^1,3^, Sven Strebel^1,4,5^, Salome Christen^6^, Eva Hägler-Laube^7^, Jochen Rössler^8^, Claudia E Kuehni^1,8^, Nicolas X von der Weid*^9^, Christina Schindera*^1,9^

**Affiliations**

^1^ Childhood Cancer Research Group, Institute of Social and Preventive Medicine, University of Bern, Switzerland

^2^ Graduate School for Cellular and Biomedical Sciences, University of Bern, Switzerland

^3^ Center for Primary Care and Public Health (Unisanté), University of Lausanne, Lausanne, Switzerland

^4^ Graduate School for Health Sciences, University of Bern, Switzerland

^5^ CANSEARCH research platform in pediatric oncology and hematology, Department of Pediatrics, Gynecology and Obstetrics, University of Geneva, Geneva, Switzerland

^6^ Department of Health Sciences and Medicine, University of Lucerne, Lucerne, Switzerland

^7^ Department of Internal Medicine, Cantonal Hospital Baden, Baden, Switzerland

^8^ Pediatric Oncology, Inselspital, Bern University Hospital, University of Bern, Switzerland

^9^ Division of Pediatric Oncology/Hematology, University Children's Hospital Basel, University of Basel, Basel, Switzerland

*These authors contributed equally to this work (shared last authorship)

**Corresponding author**

Christina Schindera, Childhood Cancer Research Group, Institute of Social and Preventive Medicine, University of Bern, Mittelstrasse 43, 3012 Bern, Switzerland.

Email: [christina.schindera@unibe.ch](mailto:christina.schindera@unibe.ch)

Tel.: [+41 31 684 37 71](tel:+41%2031%20684%2035%2007)

**Supplementary Material 1:** Checklist Individual Strength subjective fatigue subscale (CIS8R). Adapted from Worm-Smeitink M et al. J Psychosom Res. 2017 [21].

********* CIS8R *********

**Checklist Individual Strength**

**Subjective Fatigue Subscale**

Instruction:

On the next page you find 8 statements. With these statements we wish to get a sense of how you have felt during the last two weeks. For example:

**I feel relaxed**

If you feel that this statement is entirely true, tick the left box; as follows:

| **yes**, that is true | **X** |  |  |  |  |  |  | **no**, that is not true |
| --- | --- | --- | --- | --- | --- | --- | --- | --- |

I feel relaxed

If you feel that this statement is not true at all, tick the right box; as follows:

| **yes**, that is true |  |  |  |  |  |  | **X** | **no**, that is not true |
| --- | --- | --- | --- | --- | --- | --- | --- | --- |

I feel relaxed

If you feel that this statement is neither "yes, that is true", nor "no, that is not true", tick the box that best reflects how you have felt.

For example, if you feel relaxed, but not very relaxed, tick one of the boxes close to "yes, that is true": as follows:

| **yes**, that is true |  |  | **X** |  |  |  |  | **no**, that is not true |
| --- | --- | --- | --- | --- | --- | --- | --- | --- |

I feel relaxed

Do not skip any statement and select only one answer per statement.

| 1. | I feel tired | **yes, that is true** |  |  |  |  |  |  |  | **no, that is not true** |
| --- | --- | --- | --- | --- | --- | --- | --- | --- | --- | --- |
|  |  |  |  |  |  |  |  |  |  |  |
| 2. | Physically, I feel exhausted | **yes, that is true** |  |  |  |  |  |  |  | **no, that is not true** |
|  |  |  |  |  |  |  |  |  |  |  |
| 3. | I feel fit | **yes, that is true** |  |  |  |  |  |  |  | **no, that is not true** |
|  |  |  |  |  |  |  |  |  |  |  |
| 4. | I feel weak | **yes, that is true** |  |  |  |  |  |  |  | **no, that is not true** |
|  |  |  |  |  |  |  |  |  |  |  |
| 5. | I feel rested | **yes, that is true** |  |  |  |  |  |  |  | **no, that is not true** |
|  |  |  |  |  |  |  |  |  |  |  |
| 6. | Physically I am in bad shape | **yes, that is true** |  |  |  |  |  |  |  | **no, that is not true** |
|  |  |  |  |  |  |  |  |  |  |  |
| 7. | I tire easily | **yes, that is true** |  |  |  |  |  |  |  | **no, that is not true** |
|  |  |  |  |  |  |  |  |  |  |  |
| 8. | Physically I feel I am in good shape | **yes, that is true** |  |  |  |  |  |  |  | **no, that is not true** |

SCORING CIS8R

For the items: 3, 5, 8 is the scoring as follows:

| **yes**, that is true | **1** | **2** | **3** | **4** | **5** | **6** | **7** | **no**, that is not true |
| --- | --- | --- | --- | --- | --- | --- | --- | --- |

For the items: 1, 2, 4, 6, 7 is the scoring as follows:

| **yes**, that is true | **7** | **6** | **5** | **4** | **3** | **2** | **1** | **no**, that is not true |
| --- | --- | --- | --- | --- | --- | --- | --- | --- |

Subsequently the total score is calculated by summing all items.

**Supplementary Material 2:** Numerical Rating Scale (NRS) for fatigue screening.

On the following scale: How intense / strong is your fatigue at the moment?

| Not fatigued at all | 0 | 1 | 2 | 3 | 4 | 5 | 6 | 7 | 8 | 9 | 10 | Completely fatigued |
| --- | --- | --- | --- | --- | --- | --- | --- | --- | --- | --- | --- | --- |

**Supplementary Figure 1:**  Venn diagram illustrating the relationship between CCS identified as having increased or severe CRF by CIS8R and CCS identified as moderately or severely fatigued using NRS.


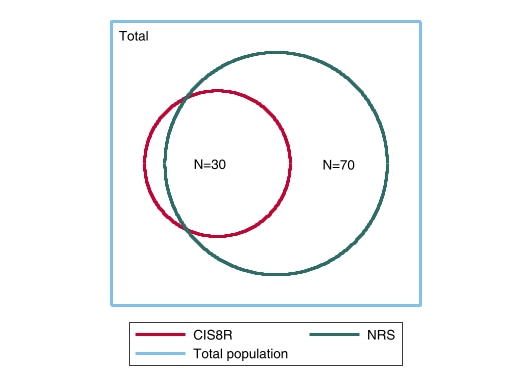


Abbreviations: CIS8R, Checklist Individual Strength subjective fatigue subscore; CRF, cancer-related fatigue; NRS, numerical rating scale

**Supplementary Table 1:** Comparison of available socio-demographic, lifestyle, clinical, and treatment-related characteristics of participating adult survivors of childhood cancer with those who participated in the CardioOnco study but did not fill out our fatigue measuring instruments, and those who were invited but did not attend the cardiooncological outpatient clinic (non-participants)

|  | Participants  N=158 (%) *^a^* | CardioOnco study participants who did not fill out fatigue instruments  N=127 (%) *^a^* | p^b^ | Non-participants  N=244 (%) *^a^* | p^c^ |
| --- | --- | --- | --- | --- | --- |
| **Socio-demographic characteristics**  Age at study, years, median [IQR]^d^  Age at study, categories^d^  < 30 years  30 – 39 years  ≥ 40 years  Married, yes  Children, yes  Employment, yes | 33 [26–38]  59 (37%)  68 (43%)  31 (20%)  53 (34%)  42 (27%)  126 (80%) | 32 [25–38]  57 (45%)  48 (38%)  22 (17%)  41 (32%)  36 (28%)  99 (78%) | 0.455  0.436  0.822  0.740  0.712 | 30 [23–38]  122 (50%)  75 (31%)  47 (19%)  -  -  - | 0.063  **0.024**  n.a.  n.a.  n.a. |
| **Lifestyle characteristics**  Smoking currently, yes  Body mass index, kg/m^2^, median [IQR]  Body mass index, categories ^e^  Underweight  Normal weight  Overweight or obese  Waist-hip ratio, median [IQR]  Waist-hip ratio, categories^f^  No abdominal obesity  Abdominal obesity present  *Missing measurement* | 24 (15%)  23.5 [21.3–26.4]  4 (3%)  95 (60%)  59 (37%)  0.83 [0.78–0.91]  97 (61%)  46 (29%)  15 (9%) | 23 (18%)  24.0 [21.3–26.8]  7 (6%)  74 (58%)  46 (36%)  0.83 [0.78–0.91]  63 (50%)  33 (26%)  31 (24%) | 0.568  0.900  0.417  0.685  0.722 | -  -  -  -  -  -  -  -  - | n.a.  n.a.  n.a.  n.a.  n.a. |
| **Clinical characteristics**  Age at diagnosis, years, median [IQR]  Age at diagnosis, categories  0 – 4 years  5 – 9 years  10 – 14 years  15 – 20 years  Time since diagnosis, years, median [IQR]  Time since diagnosis, categories  5 – 9 years  10 – 19 years  20 – 29 years  ≥ 30 years  ICCC-3 cancer diagnoses  I Leukemias  II Lymphomas  III CNS tumors  IV Neuroblastoma  V Retinoblastoma  VI Renal tumors  VII Hepatic tumors  VIII Malignant bone tumors  IX Soft tissue sarcomas  X Germ cell tumors  XI-XII Other tumors  Relapse, yes  Second primary malignancy, yes  Sleep disturbance^g^, yes  Endocrine disorder^h^, yes  Intake of antidepressants, yes | 7 [2–13]  62 (39%)  31 (20%)  47 (30%)  18 (11%)  25 [18–32]  11 (7%)  38 (24%)  52 (33%)  57 (36%)  58 (37%)  34 (22%)  8 (5%)  6 (4%)  3 (2%)  11 (7%)  2 (1%)  18 (11%)  10 (6%)  1 (1%)  7 (4%)  21 (13%)  8 (5%)  44 (28%)  35 (22%)  11 (7%) | 6 [2–12]  55 (43%)  23 (18%)  37 (29%)  12 (9%)  26 [16–32]  16 (13%)  24 (19%)  36 (28%)  51 (40%)  52 (41%)  25 (20%)  7 (6%)  6 (5%)  2 (2%)  7 (6%)  2 (2%)  10 (8%)  7 (6%)  4 (3%)  5 (4%)  12 (9%)  6 (5%)  29 (23%)  27 (21%)  10 (8%) | 0.691  0.890  0.649  0.261  0.923  0.314  0.895  0.335  0.856  0.770 | 7 [3–12]  84 (34%)  69 (28%)  64 (26%)  27 (11%)  23 [15–31]  25 (10%)  70 (29%)  85 (35%)  64 (26%)  79 (32%)  55 (23%)  35 (14%)  6 (2%)  3 (1%)  14 (6%)  4 (2%)  17 (7%)  16 (7%)  2 (1%)  13 (5%)  25 (10%)  -  -  -  - | 0.333  0.268  **0.022**  0.164  0.296  0.349  n.a.  n.a.  n.a.  n.a. |
| **Treatment-related characteristics**  Chemotherapy, yes  Anthracyclines, yes  Anthracyclines, cumulative dose  > 0 and < 250 mg/m^2^  ≥ 250mg/m^2^  Alkylating agents, yes  Radiotherapy, yes  Heart-relevant radiotherapy, yes^i^  Heart-relevant radiotherapy, cumulative dose^i^  > 0 and < 15 Gy  ≥15 and < 35 Gy  ≥ 35 Gy  Cranial radiotherapy, yes  Cranial radiotherapy, cumulative dose  > 0 and < 35 Gy  ≥ 35 Gy  Radiotherapy relevant to both brain and heart^j^  Hematopoietic stem cell transplantation, yes  Intrathoracic surgery, yes  Treatment era  1976 to 1985  1986 to 1995  1996 to 2005  2006 to 2015  Duration of treatment, months, median [IQR]^k^  Duration of treatment, categories^k^  ≤ 1 year  > 1 year | 158 (100%)  107 (68%)  67 (42%)  40 (25%)  96 (61%)  56 (35%)  46 (29%)  9/46 (6%)  20/46 (13%)  17/46 (11%)  24 (15%)  12 (8%)  12 (8%)  14 (9%)  9 (6%)  19 (12%)  35 (22%)  62 (39%)  44 (28%)  17 (11%)  12 [6–31]  79 (50%)  79 (50%) | 124 (98%)  81 (64%)  57 (45%)  24 (19%)  75 (59%)  34 (27%)  26 (20%)  5 (4%)  10 (8%)  11 (9%)  16 (13%)  7 (6%)  9 (7%)  9 (7%)  3 (2%)  16 (13%)  30 (24%)  44 (35%)  32 (25%)  21 (17%)  10.4 [4.6–31.0]  69 (54%)  58 (46%) | 0.052  0.485  0.267  0.283  0.118  0.095  0.894  0.531  0.698  0.598  0.164  0.528  0.491  0.413  0.534 | 214 (88%)  -  -  -  -  88 (36%)  -  -  -  -  -  -  -  -  4 (2%)  -  64 (26%)  85 (35%)  70 (29%)  25 (10%)  12 [5–30]  120 (49%)  124 (51%) | **<0.001**  n.a.  n.a.  n.a.  0.899  n.a.  n.a.  n.a.  n.a.  n.a.  **0.025**  n.a.  0.752  0.718  0.776 |

Abbr.: N, number; IQR, interquartile range; n.a., not available; ICCC-3, International Classification of Childhood Cancer 3rd edition; CNS, central nervous system

^a^ Column percentages are given

^b^ p-value comparing participants with those who participated in the CardioOnco study but did not fill out the fatigue measuring instruments. For categorical variables retrieved from chi-square statistics, for continuous variables retrieved from t-test.

^c^ p-value comparing participants with non-participants. For categorical variables retrieved from chi-square statistics, for continuous variables retrieved from t-test.

^d^ For non-participants calculated using March 1^st^, 2016 as the beginning of the study.

^e^ Body mass index was classified as underweight (<18.5kg/m^2^), normal weight (≥18.5 - <25kg/m2), overweight (≥25 -<30kg/m2), and obese (≥30kg/m2) [13]

^f^ Abdominal obesity was defined according to WHO cut-off point as waist-hip ratio ≥0.90 cm in men and ≥0.85 cm in women [14]

^g^ Sleep disturbance deemed to be present if survivors answered “yes” to one or more of following questions: “Do you have problems falling asleep?”, “Do you have problems sleeping through the night?” or “Do you wake up multiple times during the night?”.

^h^ Including hyperthyroidism, hypothyroidism, diabetes mellitus, diabetes insipidus, growth hormone deficiency, and any other hormonal disorder

^i^ According to the Children´s Oncology Group Guidelines Version 5.0 (i. e. chest, abdomen, whole or thoracic spine, total body irradiation) [16]

^j^ Including survivors who received total body irradiation

^k^ Including treatment of primary cancer and relapses

**Supplementary Table 2:** Regression coefficients of CRF scores (as measured by CIS subjective fatigue subscore and NRS) in adult childhood cancer survivors retrieved from multivariable linear model including sex, age at study, ICCC-3 cancer diagnoses, sleep disturbance, and endocrine disorder.

|  | **CIS subjective fatigue score**  **CIS8R** | | | **NRS score** | | |
| --- | --- | --- | --- | --- | --- | --- |
|  | β  coeff. ^a^ | 95% CI | p^b^ | β  coeff. ^a^ | 95% CI | p^b^ |
| Sex  Male  Female | Ref.  2.4 | 0.7 – 4.2 | **0.005** | Ref.  1.8 | 1.1 – 2.6 | **<0.001** |
| Age at study  < 30  30 – 39  ≥ 40 | Ref.  -2.6  -1.9 | -4.5 – -0.7  -4.3 – 0.6 | **0.023** | Ref.  -0.8  -0.8 | -1.6 - 0.0  -1.8 – 0.2 | 0.091 |
| ICCC-3 cancer diagnoses  I Leukemias  II Lymphomas  III CNS tumors  IV-XII Other | Ref.  1.9  5.3  1.7 | -0.6 – 4.3  0.7 – 9.9  -0.2 – 3.7 | 0.074 | Ref.  0.4  1.7  0.6 | -0.7 – 1.4  -0.2 – 3.6  -0.2 – 1.5 | 0.215 |
| Sleep disturbance^c^  No  Yes | Ref.  4.6 | 2.7 – 6.6 | **<0.001** | Ref.  1.4 | 0.6 – 2.2 | **<0.001** |
| Endocrine disorder^d^  No  Yes | Ref.  3.0 | 0.6 – 5.4 | **0.011** | Ref.  0.9 | -0.1 – 1.9 | 0.073 |

Abbr.: CIS8R, Checklist Individual Strength subjective fatigue subscore; CRF, cancer-related fatigue; ICCC-3, International Classification of Childhood Cancer, Third Edition; NRS, numerical rating scale; 95% CI, 95% confidence interval; p, p-value; CNS, central nervous system

^a^ β coefficient is the degree of change in the CRF score for one subgroup of patients compared to the reference subgroup.

^b^ p-value retrieved from likelihood-ratio test

^c^ Sleep disturbance deemed to be present if survivors answered “yes” to one or more of following questions: “Do you have problems falling asleep?”, “Do you have problems sleeping through the night?” or “Do you wake up multiple times during the night?”.

^d^ Including hyperthyroidism, hypothyroidism, diabetes mellitus, diabetes insipidus, growth hormone deficiency, and any other hormonal disorder

**Supplementary Table 3A:** Regression coefficients of CRF scores (as measured by CIS subjective fatigue subscore and NRS) in adult childhood cancer survivors by socio-demographic and lifestyle characteristics retrieved from univariable linear regression.

|  | **CIS subjective fatigue score**  **CIS8R** | | | | **NRS score** | | | | |
| --- | --- | --- | --- | --- | --- | --- | --- | --- | --- |
|  | β  coeff. ^a^ | 95% CI | p^b^ | | β  coeff. ^a^ | | 95% CI | | p^b^ |
| **Socio-demographic characteristics** | | | | | | | | | |
| Sex  Male  Female | Ref.  3.2 | 1.3 – 5.2 | **0.001** | | Ref.  2.1 | | 1.3 – 2.9 | | **<0.001** |
| Age at study - continuous | 0.0 | -0.2 – 0.1 | 0.431 | | 0.0 | | -0.1 – 0.0 | | 0.122 |
| Age at study  < 30  30 – 39  ≥ 40 | Ref.  -2.9  -1.0 | -5.1 – -0.7  -3.7 – 1.8 | **0.036** | | Ref.  -1.1  -0.6 | | -2.0 – -0.1  -1.7 – 0.5 | | 0.076 |
| Marriage  No  Yes | Ref.  -2.2 | -4.3 – -0.1 | **0.044** | | Ref.  -0.9 | | -1.7 – 0.0 | | **0.045** |
| Children  No  Yes | Ref.  -1.8 | -4.1 – 0.4 | 0.116 | | Ref.  -0.6 | | -1.6 – 0.3 | | 0.172 |
| Employment  No  Yes | Ref.  -3.4 | -5.9 – -1.0 | **0.006** | | Ref.  -0.9 | | -2.0 – 0.1 | | 0.072 |
| **Lifestyle characteristics** | | | | | | | | | |
| Smoking  Never  Current  Former | Ref.  1.6  -2.6 | -1.2 – 4.4  -5.7 – 0.6 | 0.111 | Ref.  0.2  -1.0 | | -0.9 – 1.4  -2.4 – 0.3 | | 0.238 | |
| Body mass index^c^ - continuous | 0.1 | -0.1 – 0.4 | 0.255 | 0.0 | | -0.1 – 0.1 | | 0.461 | |
| Body mass index^c^  Normal weight  Underweight  Overweight  Obese | Ref.  -3.4  0.9  1.1 | -9.8 – 3.1  -1.4 – 3.2  -2.4 – 4.5 | 0.550 | Ref.  -1.1  -0.3  -0.3 | | -3.7 – 1.6  -1.3 – 0.6  -1.7 – 1.1 | | 0.779 | |
| Waist-hip ratio^d^ - continuous | -10.1 | -21.9 – 1.7 | 0.093 | -8.3 | | -13.0 – -3.6 | | **<0.001** | |
| Waist-hip ratio^d^  No abdominal obesity  Abdominal obesity present | Ref.  0.1 | -2.2 – 2.4 | 0.932 | Ref.  -0.6 | | -1.5 – 0.3 | | 0.178 | |

Abbr.: CIS8R, Checklist Individual Strength subjective fatigue subscore; CRF, cancer-related fatigue; NRS, numerical rating scale; 95% CI, 95% confidence interval; p, p-value

^a^ β coefficient is the degree of change in the CRF score for one subgroup of patients compared to the reference subgroup.

^b^ p-value retrieved from likelihood-ratio test

^c^ Body mass index was classified as underweight (<18.5kg/m^2^), normal weight (≥18.5 - <25kg/m2), overweight (≥25 -<30kg/m2), and obese (≥30kg/m2) [13]

^d^ Abdominal obesity was defined according to WHO cut-off point as waist-hip ratio ≥0.90 cm in men and ≥0.85 cm in women. Patients with missing measurement of the waist-hip ratio were excluded in this analysis. [14]

**Supplementary Table 3B:** Regression coefficients of CRF scores (as measured by CIS subjective fatigue subscore and NRS) in adult childhood cancer survivors by clinical characteristics retrieved from univariable linear regression.

|  | **CIS subjective fatigue score**  **CIS8R** | | | **NRS score** | | |
| --- | --- | --- | --- | --- | --- | --- |
|  | β coeff. ^a^ | 95% CI | p^b^ | β  coeff. ^a^ | 95% CI | p^b^ |
| **Clinical characteristics** | | | | | | |
| Age at diagnosis, continuous | 0.1 | -0.1 – 0.3 | 0.178 | 0.0 | -0.1 – 0.1 | 0.473 |
| Age at diagnosis, years  0-4  5-9  10-14  15-20 | Ref.  0.4  -0.1  3.4 | -2.4 – 3.1  -2.5 – 2.4  0.0 – 6.7 | 0.223 | Ref.  -0.2  0.4  0.3 | -1.3 – 1,0  -0.6 – 1.4  -1.1 – 1.7 | 0.778 |
| Time since diagnosis, continuous | -0.1 | -0.2 – 0.0 | 0.127 | 0.0 | -0.1 – 0.0 | 0.062 |
| Time since diagnosis, years  5-9  10-19  20-29  >30 | Ref.  1.4  0.4  -0.9 | -3.0 – 5.7  -3.8 – 4.6  -5.1 – 3.2 | 0.375 | Ref.  0.4  0.7  -0.6 | -1.3 – 2.2  -1.0 – 2.4  -2.3 – 1.1 | 0.077 |
| ICCC-3 cancer diagnoses  I Leukemias  II Lymphomas  III CNS tumors  IV-XII Other | Ref.  1.9  8.8  1.5 | -0.7 – 4.5  4.2 – 13.4  -0.7 – 3.8 | **0.003** | Ref.  1.0  2.5  0.5 | -1.0 – 1.2  0.6 – 4.4  -0.5 – 1.4 | 0.075 |
| History of relapse  No  Yes | Ref.  2.2 | -0.7 – 5.2 | 0.136 | Ref.  0.2 | -1.1 – 1.4 | 0.805 |
| Second primary malignancy  No  Yes | Ref.  0.6 | -4.0 – 5.2 | 0.785 | Ref.  0.3 | -1.5 – 2.2 | 0.718 |
| Sleep disturbance^c^  No  Yes | Ref.  5.5 | 3.5 – 7.6 | **<0.001** | Ref.  1.9 | 1.0 – 2.8 | **<0.001** |
| Endocrine disorder^d^  No  Yes | Ref.  5.0 | 2.7 – 7.3 | **<0.001** | Ref.  1.5 | 0.5 – 2.4 | **0.004** |
| Intake of antidepressants  No  Yes | Ref.  3.4 | -0.5 – 7.3 | 0.090 | Ref.  1.6 | 0.0 – 3.2 | **0.048** |

Abbr.: CIS8R, Checklist Individual Strength subjective fatigue subscore; CRF, cancer-related fatigue;NRS, numerical rating scale; 95% CI, 95% confidence interval; p, p-value

ICCC-3, International Classification of Childhood Cancer, Third Edition; CNS, central nervous system

^a^ β coefficient is the degree of change in the CRF score for one subgroup of patients compared to the reference subgroup.

^b^ p-value retrieved from likelihood-ratio test

^c^ Sleep disturbance deemed to be present if survivors answered “yes” to one or more of following questions: “Do you have problems falling asleep?”, “Do you have problems sleeping through the night?” or “Do you wake up multiple times during the night?”.

^d^ Including hyperthyroidism, hypothyroidism, diabetes mellitus, diabetes insipidus, growth hormone deficiency, and any other hormonal disorder

**Supplementary Table 3C:** Regression coefficients of CRF scores (as measured by CIS subjective fatigue subscore and NRS) in adult childhood cancer survivors by treatment-related characteristics retrieved from univariable linear regression.

|  | **CIS subjective fatigue score**  **CIS8R** | | | **NRS score** | | |
| --- | --- | --- | --- | --- | --- | --- |
|  | β coeff. ^a^ | 95% CI | p^b^ | β  coeff. ^a^ | 95% CI | p^b^ |
| **Treatment-related characteristics** | | | | | | |
| Cumulative Anthracyclines, continuous | 0.0 | 0.0 – 0.0 | 0.485 | 0.0 | 0.0 – 0.0 | 0.557 |
| Cumulative Anthracyclines  No  > 0 and < 250 mg/m^2^  ≥ 250mg/m^2^ | Ref.  -1.2  1.0 | -3.5 – 1.2  -1.7 – 3.6 | 0.242 | Ref.  -0.4  0.3 | -1.4 – 0.6  -0.8 – 1.4 | 0.374 |
| Alkylating agents  No  Yes | Ref.  0.4 | -1.7 – 2.4 | 0.708 | Ref.  -0.2 | -1.0 – 0. 7 | 0.678 |
| Radiotherapy (RT)  No  Yes | Ref.  3.1 | 1.1 – 5.2 | **0.003** | Ref.  1.0 | 0.1 – 1.8 | **0.024** |
| Heart-relevant RT^c^  No  Yes | Ref.  3.3 | 1.2 – 5.5 | **0.003** | Ref.  1.2 | 0.3 – 2.1 | **0.009** |
| Heart-relevant RT^c^, continuous per 10 Gy | 1.1 | 0.4 – 1.8 | **0.002** | 0.4 | 0.1 – 0.7 | **0.009** |
| Heart-relevant RT^c^  No  > 0 and < 15 Gy  ≥15 and < 35 Gy  ≥ 35 Gy | Ref.  1.9  2.1  5.5 | -2.3 – 6.1  -0.8 – 5.1  2.3 – 8.7 | **0.006** | Ref.  1.0  0.8  1.7 | -0.8 – 2.8  -0.4 – 2.1  0.4 – 3.1 | **0.044** |
| Cranial RT  No  Yes | Ref.  3.9 | 1.1 – 6.6 | **0.006** | Ref.  0.7 | -0.5 – 1.8 | 0.231 |
| Cranial RT, continuous per 10 Gy | 1.2 | 0.5 – 1.9 | **<0.001** | 2.4 | -0.1 – 0.5 | 0.108 |
| Cranial RT  No  > 0 and < 35 Gy  ≥ 35 Gy | Ref.  0.4  7.4 | -3.3 – 4.0  3.7 – 11.0 | **<0.001** | Ref.  -0.1  1.5 | -1.7 – 1.4  0.0 – 3.1 | 0.142 |
| Cranial and/or heart-relevant RT  Neither  Heart-relevant only  Brain only  Both^d^ | Ref.  2.2  1.5  6.4 | -0.3 – 4.6  -2.5 – 5.5  3.0 – 9.9 | **0.003** | Ref.  1.0  0.0  1.6 | 0.0 – 2.0  -1.7 – 1.7  0.2 – 3.1 | 0.062 |
| Hematopoietic stem cell transplantation  No  Yes | Ref.  3.1 | -1.2 – 7.4 | 0.156 | Ref.  0.6 | -1.2 – 2.4 | 0.485 |
| Intrathoracic surgery  No  Yes | Ref.  -1.5 | -4.6 – 1.6 | 0.348 | Ref.  0.0 | -1.3 – 1.3 | 0.994 |
| Year of treatment, continuous | 0.1 | 0.0 – 0.2 | 0.150 | 0.0 | 0.0 – 0.1 | 0.098 |
| Treatment era  1976 to 1985  1986 to 1995  1996 to 2005  2006 to 2015 | Ref.  2.1  2.5  2.3 | -0.6 – 4.8  -0.3 – 5.4  -1.4 – 6.0 | 0.314 | Ref.  1.6  1.3  1.6 | 0.5 – 2.7  0.1 – 2.4  0.1 – 3.0 | **0.027** |
| Duration of treatment^e^, continuous | -1.2 | -2.1 – -0.3 | **0.013** | -0.3 | -0.7 – 0.1 | 0.106 |
| Duration of treatment^e^  ≤ 1 year  > 1 year | Ref.  -1.2 | -3.2 – 0.8 | 0.222 | Ref.  -0.3 | -1.1 – 0.5 | 0.449 |

Abbr.: CIS8R, Checklist Individual Strength subjective fatigue subscore; CRF, cancer-related fatigue; NRS, numerical rating scale; 95% CI, 95% confidence interval; p, p-value;

RT, radiotherapy

^a^ β coefficient is the degree of change in the CRF score for one subgroup of patients compared to the reference subgroup.

^b^ p-value retrieved from likelihood-ratio test

^c^ According to the COG Guidelines Version 5.0 (i. e. chest, abdomen, whole or thoracic spine, total body irradiation) [16]

^d^ Including survivors who received total body irradiation

^e^ Including treatment of primary cancer and its relapses

**Supplementary Table 4A:** Regression coefficients of CRF scores (as measured by CIS subjective fatigue subscore and NRS) in adult childhood cancer survivors retrieved from multivariable linear model including sex, age at study, ICCC-3 cancer diagnoses, sleep disturbance, and hyperthyroidism.

|  | **CIS subjective fatigue score**  **CIS8R** | | | **NRS score** | | |
| --- | --- | --- | --- | --- | --- | --- |
|  | β  coeff. ^a^ | 95% CI | p^b^ | β  coeff. ^a^ | 95% CI | p^b^ |
| Sex  Male  Female | Ref.  2.7 | 0.9 – 4.5 | **0.003** | Ref.  1.9 | 1.2 – 2.7 | **<0.001** |
| Age at study  < 30  30 – 39  ≥ 40 | Ref.  -2.6  -1.3 | -4.5 – -0.7  -3.7 – 1.2 | **0.027** | Ref.  -0.8  -0.6 | -1.6 – 0.0  -1.6 – 0.5 | 0.115 |
| ICCC-3 cancer diagnoses  I Leukemias  II Lymphomas  III CNS tumors  IV-XII Other | Ref.  2.8  8.2  2.1 | 0.4 – 5.2  4.1 – 12.3  0.1 – 4.1 | **<0.001** | Ref.  0.6  2.5  0.8 | -0.4 – 1.6  0.9 – 4.2  -0.1 – 1.6 | **0.016** |
| Sleep disturbance^c^  No  Yes | Ref.  4.7 | 2.7 – 6.7 | **<0.001** | Ref.  1.4 | 0.6 – 2.2 | **<0.001** |
| Hyperthyroidism  No  Yes | Ref.  -5.9 | -16.9 – 5.2 | 0.279 | Ref.  -2.7 | -7.2 – 1.9 | 0.237 |

Abbr.: CIS8R, Checklist Individual Strength subjective fatigue subscore; CRF, cancer-related fatigue; ICCC-3, International Classification of Childhood Cancer, Third Edition; NRS, numerical rating scale; 95% CI, 95% confidence interval; p, p-value; CNS, central nervous system

^a^ β coefficient is the degree of change in the CRF score for one subgroup of patients compared to the reference subgroup.

^b^ p-value retrieved from likelihood-ratio test

^c^ Sleep disturbance deemed to be present if survivors answered “yes” to one or more of following questions: “Do you have problems falling asleep?”, “Do you have problems sleeping through the night?” or “Do you wake up multiple times during the night?”.

**Supplementary Table 4B:** Regression coefficients of CRF scores (as measured by CIS subjective fatigue subscore and NRS) in adult childhood cancer survivors retrieved from multivariable linear model including sex, age at study, ICCC-3 cancer diagnoses, sleep disturbance, and hypothyroidism.

|  | **CIS subjective fatigue score**  **CIS8R** | | | **NRS score** | | |
| --- | --- | --- | --- | --- | --- | --- |
|  | β  coeff. ^a^ | 95% CI | p^b^ | β  coeff. ^a^ | 95% CI | p^b^ |
| Sex  Male  Female | Ref.  2.3 | 0.4 – 4.1 | **0.012** | Ref.  1.8 | 1.0 – 2.5 | **<0.001** |
| Age at study  < 30  30 – 39  ≥ 40 | Ref.  -2.6  -1.8 | -4.5 – -0.7  -4.2 – 0.7 | **0.021** | Ref.  -0.8  -0.8 | -1.6 – 0.0  -1.8 – 0.3 | 0.086 |
| ICCC-3 cancer diagnoses  I Leukemias  II Lymphomas  III CNS tumors  IV-XII Other | Ref.  2.1  5.2  1.8 | -0.4 – 4.5  0.3 – 10.2  -0.2 – 3.8 | 0.089 | Ref.  0.4  1.5  0.6 | -0.6 – 1.4  -0.5 – 3.6  -0.2 – 1.5 | 0.300 |
| Sleep disturbance^c^  No  Yes | Ref.  4.7 | 2.8 – 6.7 | **<0.001** | Ref.  1.4 | 0.6 – 2.2 | **<0.001** |
| Hypothyroidism  No  Yes | Ref.  3.0 | 0.1 – 5.8 | **0.037** | Ref.  1.00 | -0.2 – 2.2 | 0.087 |

Abbr.: CIS8R, Checklist Individual Strength subjective fatigue subscore; CRF, cancer-related fatigue; ICCC-3, International Classification of Childhood Cancer, Third Edition; NRS, numerical rating scale; 95% CI, 95% confidence interval; p, p-value; CNS, central nervous system

^a^ β coefficient is the degree of change in the CRF score for one subgroup of patients compared to the reference subgroup.

^b^ p-value retrieved from likelihood-ratio test

^c^ Sleep disturbance deemed to be present if survivors answered “yes” to one or more of following questions: “Do you have problems falling asleep?”, “Do you have problems sleeping through the night?” or “Do you wake up multiple times during the night?”.

**Supplementary Table 4C:** Regression coefficients of CRF scores (as measured by CIS subjective fatigue subscore and NRS) in adult childhood cancer survivors retrieved from multivariable linear model including sex, age at study, ICCC-3 cancer diagnoses, sleep disturbance, and diabetes mellitus.

|  | **CIS subjective fatigue score**  **CIS8R** | | | **NRS score** | | |
| --- | --- | --- | --- | --- | --- | --- |
|  | β  coeff. ^a^ | 95% CI | p^b^ | β  coeff. ^a^ | 95% CI | p^b^ |
| Sex  Male  Female | Ref.  2.6 | 0.9 – 4.4 | **0.003** | Ref.  1.9 | 1.1 – 2.6 | **<0.001** |
| Age at study  < 30  30 – 39  ≥ 40 | Ref.  -2.8  -1.7 | -4.7 – -0.9  -4.1 – 0.7 | **0.011** | Ref.  -0.9  -0.7 | -1.7 – -0.1  -1.7 – 0.3 | 0.082 |
| ICCC-3 cancer diagnoses  I Leukemias  II Lymphomas  III CNS tumors  IV-XII Other | Ref.  2.3  8.1  1.9 | -0.1 – 4.7  4.1 – 12.1  -0.1 – 3.9 | **<0.001** | Ref.  0.6  2.5  0.7 | -0.4 – 1.6  0.8 – 4.2  -0.1 – 1.5 | **0.020** |
| Sleep disturbance^c^  No  Yes | Ref.  5.0 | 3.1 – 6.9 | **<0.001** | Ref.  1.5 | 0.7 – 2.3 | **<0.001** |
| Diabetes mellitus  No  Yes | Ref.  8.6 | 2.3 – 14.9 | **0.006** | Ref.  1.6 | -1.0 – 4.2 | 0.221 |

Abbr.: CIS8R, Checklist Individual Strength subjective fatigue subscore; CRF, cancer-related fatigue; ICCC-3, International Classification of Childhood Cancer, Third Edition; NRS, numerical rating scale; 95% CI, 95% confidence interval; p, p-value; CNS, central nervous system

^a^ β coefficient is the degree of change in the CRF score for one subgroup of patients compared to the reference subgroup.

^b^ p-value retrieved from likelihood-ratio test

^c^ Sleep disturbance deemed to be present if survivors answered “yes” to one or more of following questions: “Do you have problems falling asleep?”, “Do you have problems sleeping through the night?” or “Do you wake up multiple times during the night?”.

**Supplementary Table 4D:** Regression coefficients of CRF scores (as measured by CIS subjective fatigue subscore and NRS) in adult childhood cancer survivors retrieved from multivariable linear model including sex, age at study, ICCC-3 cancer diagnoses, sleep disturbance, and diabetes insipidus.

|  | **CIS subjective fatigue score**  **CIS8R** | | | **NRS score** | | |
| --- | --- | --- | --- | --- | --- | --- |
|  | β  coeff. ^a^ | 95% CI | p^b^ | β  coeff. ^a^ | 95% CI | p^b^ |
| Sex  Male  Female | Ref.  2.7 | 0.9 – 4.5 | **0.003** | Ref.  1.9 | 1.1 – 2.6 | **<0.001** |
| Age at study  < 30  30 – 39  ≥ 40 | Ref.  -2.6  -1.6 | -4.5 – -0.7  -4.0 – 0.9 | **0.027** | Ref.  -0.8  -0.6 | -1.6 – 0.0  -1.7 – 0.4 | 0.108 |
| ICCC-3 cancer diagnoses  I Leukemias  II Lymphomas  III CNS tumors  IV-XII Other | Ref.  2.8  8.2  2.0 | 0.4 – 5.2  4.0 – 12.3  0.0 – 4.0 | **<0.001** | Ref.  0.6  2.5  0.7 | -0.4 – 1.6  0.8 – 4.2  -0.1 – 1.6 | **0.018** |
| Sleep disturbance^c^  No  Yes | Ref.  4.8 | 2.8 – 6.8 | **<0.001** | Ref.  1.4 | 0.6 – 2.2 | **<0.001** |
| Diabetes insipidus  No  Yes | Ref.  2.2 | -8.9 – 13.2 | 0.691 | Ref.  -0.7 | -5.2 – 3.9 | 0.769 |

Abbr.: CIS8R, Checklist Individual Strength subjective fatigue subscore; CRF, cancer-related fatigue; ICCC-3, International Classification of Childhood Cancer, Third Edition; NRS, numerical rating scale; 95% CI, 95% confidence interval; p, p-value; CNS, central nervous system

^a^ β coefficient is the degree of change in the CRF score for one subgroup of patients compared to the reference subgroup.

^b^ p-value retrieved from likelihood-ratio test

^c^ Sleep disturbance deemed to be present if survivors answered “yes” to one or more of following questions: “Do you have problems falling asleep?”, “Do you have problems sleeping through the night?” or “Do you wake up multiple times during the night?”.

**Supplementary Table 4E:** Regression coefficients of CRF scores (as measured by CIS subjective fatigue subscore and NRS) in adult childhood cancer survivors retrieved from multivariable linear model including sex, age at study, ICCC-3 cancer diagnoses, sleep disturbance, and growth hormone deficiency.

|  | **CIS subjective fatigue score**  **CIS8R** | | | **NRS score** | | |
| --- | --- | --- | --- | --- | --- | --- |
|  | β  coeff. ^a^ | 95% CI | p^b^ | β  coeff. ^a^ | 95% CI | p^b^ |
| Sex  Male  Female | Ref.  2.6 | 0.8 – 4.4 | **0.003** | Ref.  1.9 | 1.2 – 2.6 | **<0.001** |
| Age at study  < 30  30 – 39  ≥ 40 | Ref.  -2.5  -1.4 | -4.5 – -0.6  -3.9 – 1.1 | **0.035** | Ref.  -0.8  -0.6 | -1.6 – 0.0  -1.6 – 0.4 | 0.127 |
| ICCC-3 cancer diagnoses  I Leukemias  II Lymphomas  III CNS tumors  IV-XII Other | Ref.  2.8  7.7  2.0 | 0.4 – 5.2  2.8 – 12.6  0.0 – 4.1 | **0.004** | Ref.  0.7  2.4  0.7 | -0.3 – 1.6  0.4 – 4.4  -0.1 – 1.5 | **0.060** |
| Sleep disturbance^c^  No  Yes | Ref.  4.7 | 2.7 – 6.7 | **<0.001** | Ref.  1.4 | 0.6 – 2.2 | **<0.001** |
| Growth hormone deficiency  No  Yes | Ref.  1.0 | -4.6 – 6.6 | 0.721 | Ref.  0.3 | -2.0 – 2.6 | 0.784 |

Abbr.: CIS8R, Checklist Individual Strength subjective fatigue subscore; CRF, cancer-related fatigue; ICCC-3, International Classification of Childhood Cancer, Third Edition; NRS, numerical rating scale; 95% CI, 95% confidence interval; p, p-value; CNS, central nervous system

^a^ β coefficient is the degree of change in the CRF score for one subgroup of patients compared to the reference subgroup.

^b^ p-value retrieved from likelihood-ratio test

^c^ Sleep disturbance deemed to be present if survivors answered “yes” to one or more of following questions: “Do you have problems falling asleep?”, “Do you have problems sleeping through the night?” or “Do you wake up multiple times during the night?”.

**Supplementary Table 4F:** Regression coefficients of CRF scores (as measured by CIS subjective fatigue subscore and NRS) in adult childhood cancer survivors retrieved from multivariable linear model including sex, age at study, ICCC-3 cancer diagnoses, sleep disturbance, and other hormonal disorder.

|  | **CIS subjective fatigue score**  **CIS8R** | | | **NRS score** | | |
| --- | --- | --- | --- | --- | --- | --- |
|  | β  coeff. ^a^ | 95% CI | p^b^ | β  coeff. ^a^ | 95% CI | p^b^ |
| Sex  Male  Female | Ref.  2.8 | 1.0 – 4.6 | **0.002** | Ref.  1.9 | 1.2 – 2.7 | **<0.001** |
| Age at study  < 30  30 – 39  ≥ 40 | Ref.  -2.5  -1.4 | -4.4 – -0.6  -3.8 – 1.0 | **0.032** | Ref.  -0.8  -0.6 | -1.6 – 0.0  -1.7 – 0.4 | 0.114 |
| ICCC-3 cancer diagnoses  I Leukemias  II Lymphomas  III CNS tumors  IV-XII Other | Ref.  2.5  7.0  1.9 | 0.1 – 4.9  2.7 – 11.3  -0.1 – 3.9 | **0.005** | Ref.  0.6  2.4  0.7 | -0.4 – 1.6  0.6 – 4.1  -0.1 – 1.5 | **0.044** |
| Sleep disturbance^c^  No  Yes | Ref.  4.7 | 2.8 – 6.7 | **<0.001** | Ref.  1.4 | 0.6 – 2.2 | **<0.001** |
| Other hormonal disorder^d^  No  Yes | Ref.  2.5 | -0.5 – 5.6 | 0.086 | Ref.  0.4 | -0.9 – 1.7 | 0.517 |

Abbr.: CIS8R, Checklist Individual Strength subjective fatigue subscore; CRF, cancer-related fatigue; ICCC-3, International Classification of Childhood Cancer, Third Edition; NRS, numerical rating scale; 95% CI, 95% confidence interval; p, p-value; CNS, central nervous system

^a^ β coefficient is the degree of change in the CRF score for one subgroup of patients compared to the reference subgroup.

^b^ p-value retrieved from likelihood-ratio test

^c^ Sleep disturbance deemed to be present if survivors answered “yes” to one or more of following questions: “Do you have problems falling asleep?”, “Do you have problems sleeping through the night?” or “Do you wake up multiple times during the night?”.

^d^ Including hormonal disorders other than hyperthyroidism, hypothyroidism, diabetes mellitus, diabetes insipidus, and growth hormone deficiency
